# Bezoars as a cause of acute small bowel obstruction

**DOI:** 10.1186/1753-6561-7-S1-O1

**Published:** 2013-01-30

**Authors:** Daria Kurguzova, OP Kurguzov

**Affiliations:** 1 I.M.Sechenov First Moscow State Medical University, Moscow, Russia

## Background

Acute small bowel obstruction caused by bezoars is rare and, therefore, this problem is not well known by a community of doctors worldwide. Hence, there is high frequency of diagnostic and tactical errors and often probability of late operations. The aim of this research is to study possible reasons for the bezoar formation, clinical course and treatment of intestinal obstruction caused by bezoars.

## Methods

The present report is based on a retrospective analysis of medical records of 15 patients suffered from this form of intestinal obstruction, who were treated at the Moscow Clinical Hospital № 71 over the last 20 years. The mean age of the patients was 67.2 ± 3.4. The diagnosis was established on the basis of complaints of patients, anamnesis, results of physical examination and x-ray. 8 patients had previously undergone a gastric resection for ulcer, 1 had undergone selective proximal vagotomy and 1 had undergone stem vagotomy with pyloroplasty.

## Results

Acute small bowel obstruction caused by bezoar occurred in all 10 patients, who were operated earlier. It is associated with the fact that the above mentioned operations violate secretory and motor functions of the stomach causing the movement of poorly digested pieces of food to the small intestine and bezoar formation. Formation of bezoar is promoted by such factors as poor dental health, which does not provide proper chewing of plant products (persimmons, grapes and grapefruit) and animal products (meat), and quick swallowing, too. In the majority of patients, the course of small bowel obstruction was intermittent. All the patients were operated with diagnosis of acute intestinal obstruction. The assumption about the true nature of obstruction appeared in only 3 cases. In 10 cases bezoars were located in the ileum, and in 5 - in the jejunum. Bezoars were removed with enterotomy in 13 patients, in 2 – with fragmentation and transposition in the cecum. All operated patients have recovered.

## Conclusions

Timely diagnosis of small bowel obstruction caused by bezoar is possible by a thorough analysis of anamnestic data (information about the previous operations on the stomach, food, dental health, and character of food chewing) as well as the particular features of its clinical course, which should be intermittent. Bezoar shall be removed from enterotomy made below the obstruction, where the bowel wall microcirculatory disorders are minimal.

